# Enucleation or Hysterectomy

**Published:** 1898-04-30

**Authors:** 


### ENUCLEATION OR HYSTERECTOMY.

In a discussion on the enucleation of uterine myomata on the occasion of the last meeting of the British Gynæcological Society, the view was expressed by several speakers that the reduction of the mortality after operations for fibroids had led surgeons to operate more freely than was altogether desirable. The opinion was almost unanimous, however, that where operation was necessary, hysterectomy was a better operation than enucleation. Mr. Bland Sutton pointed out that in most cases myomata developed after the child-bearing period, and that the uterus was then a non-vital and unimportant organ. Personally he much preferred hysterectomy, leaving one or both ovaries. He felt sure that a patient was much better off with two ovaries and no uterus than with a uterus and no ovaries.

